# Supplementary material for: Genomic resources for a historical collection of cultivated two-row European spring barley genotypes
Source: Sci Data. 2024 Jan 12;11:66. doi: 10.1038/s41597-023-02850-4 (PMC10786862; doi:10.1038/s41597-023-02850-4)
Supplement: Supplementary file 1 — Supplemental Figure 1 [file 41597_2023_2850_MOESM1_ESM.docx]

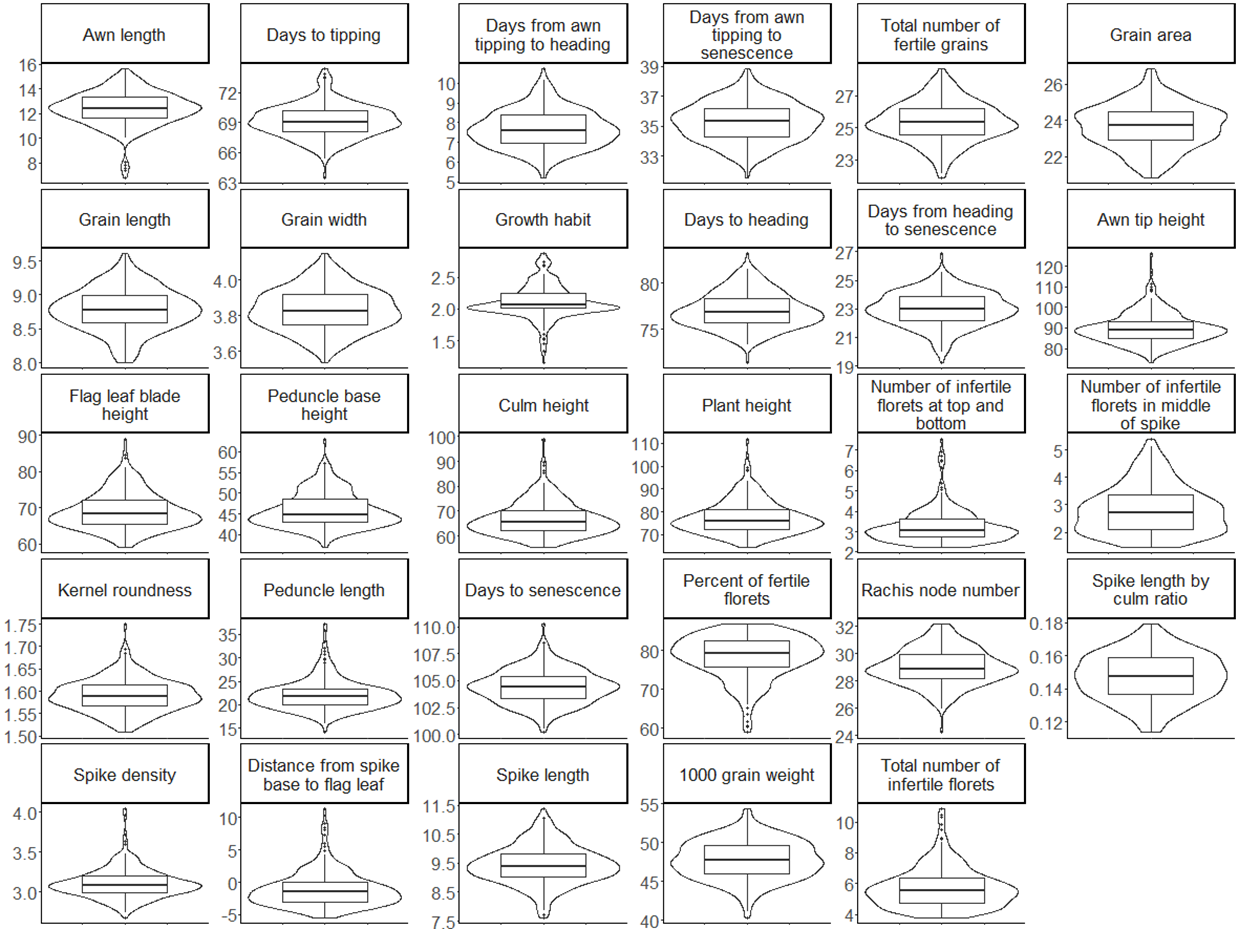


Supplemental Figure 1: Violin and boxplots of BLUP values for the phenotypic variation in all 29 scored traits.
