# Supplementary material for: Genomic resources for a historical collection of cultivated two-row European spring barley genotypes
Source: Sci Data. 2024 Jan 12;11:66. doi: 10.1038/s41597-023-02850-4 (PMC10786862; doi:10.1038/s41597-023-02850-4)
Supplement: Supplementary file 6 — Supplemental files 1 [file 41597_2023_2850_MOESM6_ESM.pdf]

```
# heliumInput = PEDIGREE
LineName Parent ParentType
Hellas Herta OTHER
Lignee 207 Herta OTHER
SW Scania Chieftain OTHER
Koral J25 OTHER
Forensic Vitesse OTHER
Cask SCRI 8313 OTHER
Tammi Olli OTHER
Vankkuri Olli OTHER
Athos Lignee 207 OTHER
Chariot Atem OTHER
Dandy Atem OTHER
Hart Atem OTHER
Stanza Atem OTHER
Senat Triple awn lemma OTHER
Baronesse (343/6 x 34/6) x J-427 OTHER
Mari Bonus OTHER
Pallas Bonus OTHER
Nackta 678 x Ub.Baco OTHER
Atlas Mutant SS 55 OTHER
Chieftain Britannia OTHER
Beka Bethges III OTHER
Bethges XIII Bethges III OTHER
Arivat Vaughn OTHER
Alsa Kneifel OTHER
Beka Kneifel OTHER
Celechovicky hanacky Kneifel OTHER
Donaria Kneifel OTHER
Rapid Kneifel OTHER
Valticky Kneifel OTHER
Brahms Acclaim OTHER
Rainbow Blenheim OTHER
Pirol Morgenrot OTHER
Piroline Morgenrot OTHER
Chevalier Tystofte 2 landvariety OTHER
Goldmarker Midas OTHER
Acoustic NSL07-8124-A OTHER
Renaissance NSL07-8124-A OTHER
Olympus Tesla OTHER
Oriol Heine 05530 OTHER
Tern Heine 05530 OTHER
Haha Heines Hanna OTHER
Haisa Heines Hanna OTHER
Svalof Hanna Weib. 5973 x Weib. 5853 OTHER
Cilla Hadostreng OTHER
Annabell Henni OTHER
Tocada Henni OTHER
Viskosa Henni OTHER
Prolog Meltan OTHER
Krystal Koral OTHER
Dray Juno OTHER
Elbo Drost OTHER
Stendes Drost OTHER
Drake Abed 3371 OTHER
```

Universe Abed 3371 OTHER
Bogart Appaloosa OTHER
Olympus Genie OTHER
Cocktail Linden OTHER
Doyen Linden OTHER
Novello Linden OTHER
Velvet Linden OTHER
Galina Ho. 4595/51 OTHER
Arivat Atlas USA OTHER
Sherpa Maris Mink OTHER
Orbit Sk 783 OTHER
Invictus Zeppelin OTHER
Swallow Donaria OTHER
Union Donaria OTHER
Luke Crusader OTHER
Spire Crusader AFP1290 OTHER
Karat B 2145 OTHER
Lada Hadm. 46619-68 OTHER
Krystal Rapid OTHER
Prodigal Varberg OTHER
Tweed Maris Mink OTHER
Vega Kristina OTHER
Delta Hordeum laevigatum OTHER
Minerva Hordeum laevigatum OTHER
Vada Hordeum laevigatum OTHER
Emir Delta OTHER
Julia Delta OTHER
Okos Stanka OTHER
Scandium Roxana OTHER
SW Scania SW 89692 OTHER
Columbus Isabella OTHER
Natasia Isabella OTHER
Imidis SJ 203256 OTHER
Maris Mink Swallow OTHER
Impala Imperial OTHER
Fairytale Power OTHER
Isabella Power OTHER
Berwick Cooper OTHER
Brazil Cooper OTHER
Century Cooper OTHER
Chalice Cooper OTHER
Hind Cooper OTHER
Columbus Publican OTHER
Gamma-Ray Mutant Maythorpe OTHER
Golden Promise Maythorpe OTHER
Triumph S3170/Abyss OTHER
Katy Arivat OTHER
Amsel Haisa OTHER
Celechovicky hanacky Haisa OTHER
Impala Haisa OTHER
Oriol Haisa OTHER
Stanka Haisa OTHER
Tern Haisa OTHER
Villa Haisa OTHER
Volla Haisa OTHER
Midas Gamma-Ray Mutant OTHER

Nomad Kym OTHER
Scarlett Kym OTHER
Abacus Vada OTHER
Alva Vada OTHER
Egmont Vada OTHER
Georgie Vada OTHER
Lud Vada OTHER
Luke Vada OTHER
Salka Vada OTHER
Universe Vada OTHER
Astrix Ares OTHER
Klaxon RPB 16/71 OTHER
Orthega Golf OTHER
Dram Tern OTHER
Koral Hana OTHER
Tankard Heritage OTHER
Alexis Breun 1622 OTHER
Libelle Breun 1622 OTHER
Meltan D 80-20 OTHER
Libelle Breun 1747 OTHER
Simba Otira OTHER
Varberg Orthega OTHER
Invictus Tamtam OTHER
RGT Conquest Tamtam OTHER
RGT Planet Tamtam OTHER
Scarlett Amazone OTHER
Atem PP79 OTHER
Avalon Beatrix OTHER
Colada NFC1983-27 OTHER
Concerto Minstrel OTHER
Senat Hellas OTHER
Fergie Athos OTHER
Regent Athos OTHER
Tyne Athos OTHER
Waggon NFC 499-69 OTHER
Dallas Sherpa OTHER
Cilla Ingrid OTHER
Sandra Ingrid OTHER
Wing Ingrid OTHER
Fleet Claret OTHER
Regatta Claret OTHER
Tennis Claret OTHER
Natasia NFC Tipple OTHER
Propino NFC Tipple OTHER
Orbit Ce Dc-74 OTHER
Sandra Beka OTHER
Flare Luke OTHER
Golf Luke OTHER
Trebon K 829 x Ariel OTHER
Aufis Wisa OTHER
Impala Wisa OTHER
Julia Wisa OTHER
Villa Wisa OTHER
Volla Wisa OTHER
Digger Universe OTHER
Magnum Universe OTHER

Sherpa Universe OTHER
Rainbow DN7 OTHER
Arvo Helmi OTHER
Skittle Ortoli OTHER
Karat 1293/70 OTHER
KWS Irina Conchita OTHER
KWS Orphelia Conchita OTHER
Amazone Aufis OTHER
Derkado Lada OTHER
Platoon Lada OTHER
Grit Hadm. 46459-68 OTHER
Lada Hadm. 46459-68 OTHER
Maresi Hadm. 46459-68 OTHER
TS42/3/5 BH4/200/5/90 OTHER
Otto African landvariety OTHER
Avalon Marnie OTHER
Aluminium Landlord OTHER
Colada Landlord OTHER
Static Landlord OTHER
Renata 26744/84 OTHER
Maypole Extract OTHER
Novello Extract OTHER
Troon Extract OTHER
Orthega Cebeco 7931 x Pompadour OTHER
SY Universal Henley OTHER
Gitane CI 1237 OTHER
Maresi Cebeco 6801 x GB 1605 OTHER
Landlord Platoon OTHER
Pitcher Platoon OTHER
Trinity Platoon OTHER
Proctor Plumage Archer OTHER
Marion Berenice OTHER
Fergie Hood OTHER
Troon NSL 95-2949 OTHER
Corniche S-487 OTHER
Prodigal Tartan OTHER
Abed Denso Rigel OTHER
Cask Fleet OTHER
Chad Fleet OTHER
Cork Fleet OTHER
Orcivale Fleet OTHER
Platoon Fleet OTHER
Nackta Peroga OTHER
Rummy Cellar OTHER
Goldie Panda OTHER
Pasadena Marina OTHER
Marina HVS 2.1142-4-79 OTHER
Piccolo Miln 155/38 OTHER
Blenheim Egmont OTHER
Dandy Egmont OTHER
Hart Egmont OTHER
Stanza Egmont OTHER
Potter 88513 OTHER
Hind Hart OTHER
Juno Hart OTHER
Linden Hart OTHER

Invictus Columbus OTHER
Centurion Saloon OTHER
Cocktail Saloon OTHER
Power Saloon OTHER
Armelle Clermont OTHER
Corgi 15533 Co OTHER
Zephyr 2RSB Heine 2149 OTHER
Macaw Dray OTHER
Novello Dray OTHER
Scarlett Lignee 2730E OTHER
Tartan Grand Prix 2 OTHER
SY Universal Propino OTHER
Hellas Pallas OTHER
Rupal Pallas OTHER
Senat Pallas OTHER
Rupee Indian landrace OTHER
Tammi Asplund OTHER
Vankkuri Asplund OTHER
Roxana Korinna OTHER
Ria Hadm. 55648-85 OTHER
Cropton Waggon OTHER
Gunilla Birgitta OTHER
Salve Birgitta OTHER
Cork Peel OTHER
Dera Galina OTHER
Salome Galina OTHER
Maud V 813 OTHER
Bartok Apex OTHER
Britannia Apex OTHER
Heritage Apex OTHER
Reggae Apex OTHER
Saxo Apex OTHER
TSS 311-54 Apex OTHER
Balder Scanian Barley OTHER
Barke Libelle OTHER
Atlas Diamant OTHER
Camargue Diamant OTHER
Corniche Diamant OTHER
Favorit Diamant OTHER
Hana Diamant OTHER
Karat Diamant OTHER
Rapid Diamant OTHER
Spartan Diamant OTHER
Triumph Diamant OTHER
Apex Julia OTHER
Marnie Breun 4714 OTHER
Prague CPBT B55 OTHER
Steina Sultan OTHER
Abacus Zephyr 2RSB OTHER
Atem Zephyr 2RSB OTHER
Georgie Zephyr 2RSB OTHER
Gitane Zephyr 2RSB OTHER
Lud Zephyr 2RSB OTHER
Abava Elsa OTHER
Tremois Dram OTHER
Kassima Orcivale OTHER

Concerto Westminster OTHER
Regatta PF 52213 OTHER
Drum Trinity OTHER
Balga KM 1192 OTHER
Camargue KM 1192 OTHER
Apex Volla OTHER
Aramir Volla OTHER
Drake Volla OTHER
Galina Volla OTHER
Steina Volla OTHER
Villa Volla OTHER
Amsel Pirol OTHER
Oriol Pirol OTHER
Tern Pirol OTHER
Baronesse LBW6153 P40 OTHER
Otira SJ 930331 OTHER
Acclaim Hadm. 46813-68 OTHER
Gerlinde Hadm. 46813-68 OTHER
Akka Arla OTHER
Velvet Berwick OTHER
TSS 311-54 TS42/3/5 OTHER
Etna Magnum OTHER
Tyne Magnum OTHER
Bethges XIII Bethges II OTHER
Peel VSB 10-15 x NFC 1440/80 OTHER
Ortoli Monarch OTHER
Elsa Maga OTHER
Romi Abed 079 OTHER
Dina Deba Abed OTHER
Maris Mink Deba Abed OTHER
22746Co41 Digger OTHER
Acoustic Cropton OTHER
KWS Vitara Cropton OTHER
Renaissance Cropton OTHER
Beatrix Viskosa OTHER
Conchita Viskosa OTHER
Sebastian Viskosa OTHER
Cellar NFC 94-11 OTHER
Chaser NFC 94-11 OTHER
Pewter NFC 94-11 OTHER
Vega Lofa OTHER
Domen Maskin OTHER
Avec Saxo OTHER
Imidis Fabel OTHER
Arla Opal OTHER
Birgitta Opal OTHER
Bonus Opal OTHER
Britta Opal OTHER
Freja Opal OTHER
Gunilla Opal OTHER
Ingrid Opal OTHER
Opal B Opal OTHER
Tellus Opal OTHER
Ymer Opal OTHER
Crusader AFP1290 Graphic OTHER
Elbo Rika OTHER

Elsa Rika OTHER
Avalon Pasadena OTHER
Beatrix Pasadena OTHER
Tocada Pasadena OTHER
Amsel Maja OTHER
Arla Maja OTHER
Balder Maja OTHER
Birgitta Maja OTHER
Bonus Maja OTHER
Carlsberg Maja OTHER
Drost Maja OTHER
Lenta Maja OTHER
Maythorpe Maja OTHER
Oriol Maja OTHER
Rigel Maja OTHER
Stendes Maja OTHER
Tern Maja OTHER
Ymer Maja OTHER
Arla Tammi OTHER
Macaw Fractal OTHER
Arla Svanhals OTHER
Lignee 207 Svanhals OTHER
Impala H 204 OTHER
Imidis Vortex OTHER
NFC Tipple Vortex OTHER
Skittle Vortex OTHER
Waggon Vortex OTHER
Oxbridge Tavern OTHER
Prague Tavern OTHER
Braemar NFC 94-20 OTHER
Cellar NFC 94-20 OTHER
Chaser NFC 94-20 OTHER
Drum NFC 94-20 OTHER
Pewter NFC 94-20 OTHER
Tavern NFC 94-20 OTHER
Vortex NFC 94-20 OTHER
Carlsberg Prentice OTHER
Rapid Voldagsen OTHER
Steina Voldagsen OTHER
Renata 22114/84M OTHER
Havanna Breun 3556a OTHER
Roxana Breun 3556a OTHER
Goldie ET 181 x Kara x Halcyon OTHER
Crusader AFP1290 Fergie OTHER
Piccolo Drake OTHER
Chime 92.25 OTHER
KWS Vitara Steward x NFC 406/113 OTHER
Katy Astrix OTHER
Denar Bavaria OTHER
Isaria Bavaria OTHER
Spartan Ekonom OTHER
Lignee 207 CJV 1483 OTHER
Agio Kenia OTHER
Ares Kenia OTHER
Berenice Kenia OTHER
Britta Kenia OTHER

Delta Kenia OTHER
Drost Kenia OTHER
Ekonom Kenia OTHER
Elsa Kenia OTHER
Emir Kenia OTHER
Herta Kenia OTHER
Lenta Kenia OTHER
Proctor Kenia OTHER
Rigel Kenia OTHER
Rika Kenia OTHER
Sandra Kenia OTHER
Sultan Kenia OTHER
Anais 413287 OTHER
Natasia Picnic OTHER
Rhynchostar Picnic OTHER
Heron Stanza OTHER
Riviera Stanza OTHER
Domen Opal B OTHER
Panda Gerbel OTHER
Fergie Goldmarker OTHER
Fleet Goldmarker OTHER
TS42/3/5 Goldmarker OTHER
Tyne Goldmarker OTHER
Tyne Goldmarker OTHER
Bogart SJ 050549 OTHER
Genie NSL04-4299-B OTHER
Aapo Riegel OTHER
Ares Bordia OTHER
Berenice Bordia OTHER
Ceres Bordia OTHER
Clermont Bordia OTHER
Casino Georgie OTHER
Kym Georgie OTHER
Regent Georgie OTHER
Donaria Isaria OTHER
Georgine Isaria OTHER
Haisa Isaria OTHER
Herta Isaria OTHER
Peroga Isaria OTHER
Rika Isaria OTHER
Weihenstephaner MR 2 Isaria OTHER
Wisa Isaria OTHER
Spey Fergie OTHER
Bartok Joline OTHER
Berenice Union OTHER
Favorit Union OTHER
Galina Union OTHER
Karat Union OTHER
Koral Union OTHER
Krona Union OTHER
Triumph Union OTHER
Berwick Riviera OTHER
Otira Bartok OTHER
Spey Woodcock OTHER
Sandra Sarah OTHER
Kassima Delibes OTHER

Scandium Delibes OTHER
Panda Katy OTHER
Starlight Z90-552 OTHER
Casino Regent OTHER
Casino Hordeum deficiens x Sergeant OTHER
Decanter Dallas OTHER
Emir Arabische OTHER
Sultan Arabische OTHER
Appaloosa 49113-502-11 OTHER
Elsa Lome OTHER
Class Optic OTHER
Drum Optic OTHER
Spire Optic OTHER
Helmi Pikkio OTHER
Conchita LP 629.1-95 OTHER
Marnie Havanna OTHER
Monte Cristo Indian landvariety OTHER
Poker SJ 96-1441 OTHER
NFC Tipple NFC 497-12 OTHER
Claret Abacus OTHER
Henni 84160.1.3.3 OTHER
Sherpa F 784-70/3 OTHER
Dera 1208/67 OTHER
Rummy Cocktail OTHER
Acclaim Triumph OTHER
Alexis Triumph OTHER
Alis Triumph OTHER
Amazone Triumph OTHER
Ariel Triumph OTHER
Blenheim Triumph OTHER
Carnival Triumph OTHER
Corgi Triumph OTHER
Cork Triumph OTHER
Dera Triumph OTHER
Gerlinde Triumph OTHER
Krona Triumph OTHER
Natasha Triumph OTHER
Nomad Triumph OTHER
Prisma Triumph OTHER
Goldthorpe Chevalier Tystofte 2 OTHER
Denar Celechovicky hanacky OTHER
Koral Celechovicky hanacky OTHER
Claret HP 5466 OTHER
Hood HP 5466 OTHER
Midas Wong OTHER
Berac Erica OTHER
Arla Svalof Hanna OTHER
Hannchen Svalof Hanna OTHER
Kym Svalof Hanna OTHER
Varberg LP 426.92 x LP 6800.92 OTHER
Defra Karat OTHER
Orthega S77323 OTHER
Magnum Magnif 104 OTHER
Ceres Piroline OTHER
Swallow Piroline OTHER
Camargue 49428/69 OTHER

Etna Alis OTHER
Paloma Alis OTHER
Digger Magnif 105 OTHER
Alis Rosie Abed OTHER
Apex Cebeco 6721 x L100 OTHER
Krona Gimpel OTHER
Deba Abed Abed Denso OTHER
22746Co41 Dera OTHER
Chariot Dera OTHER
Cork Dera OTHER
Fairytale Colston OTHER
Poker Colston OTHER
Saloon Hind OTHER
Tavern NFC 94-4 OTHER
Gitane Cambrinus OTHER
Prisma Cambrinus OTHER
Abava Domen OTHER
Kristina Domen OTHER
Centurion Class OTHER
Aluminium PF14 OTHER
Union Firl. 621 OTHER
Goldmarker TCE 141 OTHER
Steina St434/62 OTHER
Akka Monte Cristo OTHER
Elsa Monte Cristo OTHER
Grand Prix 2 Monte Cristo OTHER
Mona Monte Cristo OTHER
Spartan Monte Cristo OTHER
Svalof Hanna Monte Cristo OTHER
Sherpa HB 820-12 OTHER
Amber Force OTHER
Cooper Force OTHER
Optic Force OTHER
Platoon Force OTHER
Riviera Cebeco 8331 OTHER
Svalof Hanna Clara OTHER
Troubadour MGH 6271 OTHER
Forensic Oxbridge OTHER
Dana Ymer OTHER
Abava Mari OTHER
Kristina Mari OTHER
Mona Mari OTHER
Salve Mari OTHER
Agio Georgine OTHER
Maythorpe Goldthorpe OTHER
Steffi Stamm 210 OTHER
Dram MGH 61229 OTHER
Troubadour MGH 61229 OTHER
Tankard CSBA 1096/1022 OTHER
Derkado Salome OTHER
Marina Salome OTHER
Viskosa Salome OTHER
Prolog Etna OTHER
Alliot Alexis OTHER
Anais Alexis OTHER
Barke Alexis OTHER

Paloma Alexis OTHER
Roxana Alexis OTHER
Claret Proctor OTHER
Gimpel Proctor OTHER
Hood Proctor OTHER
Lofa Proctor OTHER
Mala Abed Proctor OTHER
Midas Proctor OTHER
Livet 22746Co41 OTHER
Triumph 11719/59 OTHER
Defra Gerlinde OTHER
Lux Goldie OTHER
Saxo HVS 18707 OTHER
Amsel Lyallpur OTHER
Oriol Lyallpur OTHER
Tern Lyallpur OTHER
Wing Lyallpur OTHER
Alabama ML-I x LP2.51784 OTHER
Steffi Stamm 101 OTHER
Fergie Marion OTHER
Oxbridge Chime OTHER
Publican Sebastian OTHER
Quench Sebastian OTHER
Marion Sandra OTHER
Erna Berac OTHER
Sherpa Berac OTHER
Tremois Berac OTHER
Armelle Ceres OTHER
Katy Ceres OTHER
Ria Hadm. 96677-87 OTHER
Salome 36462 x 14008/64 OTHER
Dray Amber OTHER
Extract Amber OTHER
Linden Amber OTHER
Egmont Maris Yak x W 1001 OTHER
Bartok Flute OTHER
Chad Natasha OTHER
Braemar NFC 5563 OTHER
Vortex NFC 5563 OTHER
Fairytale Recept OTHER
Isabella Recept OTHER
Cambrinus Streng Franken III OTHER
Tellus Streng Franken III OTHER
Gull Gotland landvariety OTHER
Brazil Trebon OTHER
Goldie Ariel OTHER
Trebon Ariel OTHER
Taphouse Braemar OTHER
Amazone Breun 1453e16 x Him.T253 OTHER
Balder Gull OTHER
Delta Gull OTHER
Gull Mutant Gull OTHER
Kenia Gull OTHER
Maja Gull OTHER
Minerva Gull OTHER
Opal Gull OTHER

Seger Gull OTHER
Vada Gull OTHER
Paloma Corgi OTHER
Alabama Krona OTHER
Annabell Krona OTHER
Hadm. 96677-87 Krona OTHER
Pasadena Krona OTHER
Alva Balder OTHER
Arvo Balder OTHER
Balder J Balder OTHER
Berac Balder OTHER
Britta Balder OTHER
Cambrinus Balder OTHER
Impala Balder OTHER
Ingrid Balder OTHER
Sultan Balder OTHER
Tellus Balder OTHER
Appaloosa Decanter OTHER
Reggae VDH 233-79 OTHER
Nordal Dana OTHER
Salome Hadm. 46655 OTHER
Rapid Denar OTHER
Goldie Tellus OTHER
Meltan Tellus OTHER
Simon Tellus OTHER
Hood Dwarf OTHER
Natasia Scandium OTHER
Balga Gunilla OTHER
Carnival Maris Bulbeck OTHER
Husky AFP2429 SJ 028126 OTHER
Orcivale Defra OTHER
Georgine Moosburger Rhatia OTHER
Gimpel Carlsberg II OTHER
Koral Carlsberg II OTHER
Tweed Akka OTHER
Tesla Vivaldi OTHER
Westminster Barke OTHER
Berenice Frisia OTHER
Clermont Frisia OTHER
Haha Hado OTHER
Amber Chad OTHER
Optic Chad OTHER
Chad NFC 461181 OTHER
Dina Amsel OTHER
Sandra Amsel OTHER
Extract Cask OTHER
Astrix Hauter x Hatif de Grignon OTHER
Joline Klaxon OTHER
Alva Binder OTHER
Britta Binder OTHER
Helmi Binder OTHER
Ingrid Binder OTHER
Kenia Binder OTHER
Maja Binder OTHER
Opal Binder OTHER
Tellus Binder OTHER

Ariel All 3109 OTHER
SY Taberna Taphouse OTHER
Dallas Grit OTHER
Dera Grit OTHER
Betzes Bethges XIII OTHER
Tennis Erna OTHER
Taphouse Wicket OTHER
Alva Seger OTHER
Bonus Seger OTHER
Freja Seger OTHER
Ymer Seger OTHER
Okos Otto OTHER
Okos Otto OTHER
Seger Hannchen OTHER
Avec Maud OTHER
Kassima Maud OTHER
Potter Maud OTHER
Gimpel Stamm OTHER
Havanna 3192 f 56 OTHER
Simba Prolog OTHER
Century Brahms OTHER
Salka Elbo OTHER
Libelle Rupee OTHER
Rupal Rupee OTHER
Apex Aramir OTHER
Digger Aramir OTHER
Erna Aramir OTHER
Grand Prix 2 Aramir OTHER
Natasha Aramir OTHER
Steffi Aramir OTHER
Tremois Aramir OTHER
Viskosa Maresi OTHER
Joline RPB 713/77 OTHER
Power Annabell OTHER
Prisma Piccolo OTHER
Chieftain Prisma OTHER
Marnie Prisma OTHER
Alliot Chariot OTHER
Chalice Chariot OTHER
Dray Chariot OTHER
Extract Chariot OTHER
Landlord Chariot OTHER
Linden Chariot OTHER
Ortoli Chariot OTHER
Pitcher Chariot OTHER
Prestige Chariot OTHER
Starlight Chariot OTHER
Tankard Chariot OTHER
Trinity Chariot OTHER
Cocktail Colada OTHER
Doyen Colada OTHER
Novello Colada OTHER
Power Colada OTHER
Chalice NFC 514-5 OTHER
Linden NFC 514-5 OTHER
Chariot Carnival OTHER

Gunilla Gull Mutant OTHER
Maud Flare OTHER
Isaria Danubia OTHER
Amber NFC 85/1/3 OTHER
Cask Regatta OTHER
Delibes Regatta OTHER
Peel Regatta OTHER
Class Prestige OTHER
Maypole Prestige OTHER
Peroga Sulu OTHER
Baronesse Oriol OTHER
Celechovicky hanacky Nolcuv A or Dregerev Imp OTHER
Claret Armelle OTHER
Flare Armelle OTHER
Golf Armelle OTHER
Hood Armelle OTHER
TS42/3/5 Armelle OTHER
Chime Heron OTHER
Decanter Heron OTHER
Momentum Concerto OTHER
Odyssey Concerto OTHER
Overture Concerto OTHER
RGT Conquest Concerto OTHER
RGT Planet Concerto OTHER
Henni Baronesse OTHER
Klaxon Nackta OTHER
Camargue 14029 OTHER
Corniche 14029 OTHER
Triumph 14029 OTHER
Birgitta Vega OTHER
Gunilla Vega OTHER
Valticky Valticky B OTHER
Deba Abed Weihenstephaner MR 2 OTHER
Peroga Weihenstephaner MR 2 OTHER
Piroline Weihenstephaner MR 2 OTHER
Union Weihenstephaner MR 2 OTHER
Midas Weihenstephaner MR 1 OTHER
Pirol Weihenstephaner MR 1 OTHER
Stanka Weihenstephaner MR 1 OTHER
Weihenstephaner MR 2 Weihenstephaner MR 1 OTHER
Wisa Weihenstephaner MR 1 OTHER
Hana Alsa OTHER
Koral Alsa OTHER
Triumph Alsa OTHER
Bogart Quench OTHER
Cropton Quench OTHER
Genie Quench OTHER
Husky AFP2429 Quench OTHER
KWS Irina Quench OTHER
KWS Orphelia Quench OTHER
KWS Orphelia Quench OTHER
Momentum Quench OTHER
Odyssey Quench OTHER
Overture Quench OTHER
Propino Quench OTHER
Rhynchostar Quench OTHER

Summit Quench OTHER
SY Taberna Quench OTHER
Tamtam Quench OTHER
Tesla Quench OTHER
Gimpel Heine 4808 OTHER
Nordal Heine 4808 OTHER
Aramir Emir OTHER
Atem Emir OTHER
Athos Emir OTHER
Corniche Emir OTHER
Maris Mink Emir OTHER
Landlord NFC86/60 OTHER
Amber Corniche OTHER
Cooper Corniche OTHER
Delibes Corniche OTHER
Heron Corniche OTHER
Juno Corniche OTHER
Krona Corniche OTHER
Optic Corniche OTHER
Tankard Corniche OTHER
Summit Tocada OTHER
Binder Proskowetz Hanna OTHER
Binder Abed Proskowetz Hanna OTHER
Kneifel Proskowetz Hanna OTHER
Flare Lud OTHER
Golf Lud OTHER
Diamant Valticky OTHER
Ekonom Valticky OTHER
Karat Valticky OTHER
Spartan Valticky OTHER
Westminster NSL 97-5547 OTHER
Atem Minerva OTHER
Lofa Minerva OTHER
Mala Abed Minerva OTHER
Bavaria Bavarian landvariety OTHER
Romi Rupal OTHER
Cellar Cork OTHER
Chaser Cork OTHER
Lux Cork OTHER
NFC Tipple Cork OTHER
Prestige Cork OTHER
Saloon Cork OTHER
Static Cork OTHER
Forensic Troon OTHER
SY Universal Troon OTHER
Publican Drum OTHER
Quench Drum OTHER
Cooper Troop OTHER
Livet TSS 311-54 OTHER
Grit Hadm. 55474-67 x Derenburg 480-68 OTHER
Power Lux OTHER
Sebastian Lux OTHER
Tartan Golden Promise OTHER
Alsa Haha OTHER
Emir Agio OTHER
Sultan Agio OTHER

Brahms Casino OTHER
Britannia Casino OTHER
Heritage Casino OTHER
Aapo Carlsberg OTHER
Aufis Carlsberg OTHER
Carlsberg II Carlsberg OTHER
Dana Carlsberg OTHER
Karat Carlsberg OTHER
Nackta Carlsberg OTHER
Sandra Carlsberg OTHER
Steina Carlsberg OTHER
Zephyr 2RSB Carlsberg OTHER
Peroga Ragusa OTHER
Stanka Ragusa OTHER
Delta AFP460 Tyra OTHER
Delta AFP460 Claret OTHER
Tyra Algerian x Herta 8 OTHER
Tyra Rika OTHER
Tyra Drost OTHER
Crusader Earl OTHER
Crusader Heine 4242 OTHER
Crusader Rika OTHER
