# Supplementary material for: Genomic resources for a historical collection of cultivated two-row European spring barley genotypes
Source: Sci Data. 2024 Jan 12;11:66. doi: 10.1038/s41597-023-02850-4 (PMC10786862; doi:10.1038/s41597-023-02850-4)
Supplement: Supplementary file 7 — Supplemental files 2 [file 41597_2023_2850_MOESM7_ESM.pdf]

heliumInput = PHENOTYPE
germplasm_name germplasm_id germplasm_gid country_name country_code has_trials_data has_genotypic_data Project Year Range
Aapo 1 Aapo Finland FI 1 1 BARN 1975 1970-1979
Abacus 2 Abacus United Kingdom of Great Britain and Northern Ireland GB 1 1 BARN 1974 1970-1979
Abava 3 Abava Latvia LV 1 1 BARN 1978 1970-1979
Acoustic 4 Acoustic United Kingdom of Great Britain and Northern Ireland GB 1 1 BARN 2011 2010-2017
Alabama 5 Alabama Germany DE 1 1 BARN 1998 1990-1999
Alexis 6 Alexis Germany DE 1 1 BARN 1986 1980-1989
Alliot 7 Alliot Denmark DK 1 1 BARN 1998 1990-1999
Aluminium 8 Aluminium Denmark DK 1 1 BARN 2003 2000-2009
Alva 9 Alva Sweden SE 1 1 BARN 1977 1970-1979
Anais 10 Anais Germany DE 1 1 BARN 1999 1990-1999
Annabell 11 Annabell Germany DE 1 1 BARN 1997 1990-1999
Apex 12 Apex Netherlands NL 1 1 BARN 1983 1980-1989
Appaloosa 13 Appaloosa United Kingdom of Great Britain and Northern Ireland GB 1 1 BARN 2003 2000-2009
Aramir 14 Aramir Netherlands NL 1 BARN 1972 1970-1979
Armelle 15 Armelle France FR 1 1 BARN 1974 1970-1979
Arvo 16 Arvo Finland FI 1 1 BARN 1966 1960-1969
Athos 17 Athos France FR 1 1 BARN 1975 1970-1979
Atlas 18 Atlas Czech Republic CZ 1 1 BARN 1976 1970-1979
Avalon 19 Avalon Germany DE 1 1 BARN 2011 2010-2017
Avec 20 Avec Sweden SE 1 1 BARN 1995 1990-1999
Balder 21 Balder Sweden SE 1 1 BARN 1942 1830-1959
Balder J 22 Balder J Sweden SE 1 BARN 1964 1960-1969
Balga 23 Balga Latvia LV 1 1 BARN 1990 1990-1999
Barke 24 Barke Germany DE 1 1 BARN 1996 1990-1999
Baronesse 25 Baronesse Germany DE 1 1 BARN 1989 1980-1989
Beatrix 26 Beatrix Germany DE 1 1 BARN 2003 2000-2009
Beka 27 Beka France FR 1 1 BARN 1954 1830-1959
Berac 28 Berac Netherlands NL 1 1 BARN 1969 1960-1969
Berenice 29 Berenice France FR 1 1 BARN 1972 1970-1979
Berwick 30 Berwick United Kingdom of Great Britain and Northern Ireland GB 1 1 BARN 1997 1990-1999
Betzes 31 Betzes Germany DE 1 1 BARN 1957 1830-1959
Binder 32 Binder Denmark DK 1 1 BARN 1913 1830-1959
Binder Abed 33 Binder Abed Denmark DK 1 1 BARN 1913 1830-1959
Birgitta 34 Birgitta Sweden SE 1 1 BARN 1963 1960-1969
Blenheim 35 Blenheim United Kingdom of Great Britain and Northern Ireland GB 1 1 BARN 1984 1980-1989
Bogart 36 Bogart United Kingdom of Great Britain and Northern Ireland GB 1 1 BARN 2009 2000-2009
Bonus 37 Bonus Sweden SE 1 1 BARN 1950 1830-1959
Braemar 38 Braemar United Kingdom of Great Britain and Northern Ireland GB 1 1 BARN 1999 1990-1999
Brazil 39 Brazil France FR 1 1 BARN 2000 2000-2009
Britta 40 Britta Sweden SE 1 1 BARN 1964 1960-1969
Camargue 41 Camargue Germany DE 1 1 BARN 1983 1980-1989
Campala 42 Campala France FR 1 1 BARN 2000 2000-2009
Carlsberg 43 Carlsberg Denmark DK 1 1 BARN 1946 1830-1959
Cellar 44 Cellar United Kingdom of Great Britain and Northern Ireland GB 1 1 BARN 1998 1990-1999

Centurion 45 Centurion United Kingdom of Great Britain and Northern Ireland GB 1 1 BARN 1996 1990-1999
Century 46 Century United Kingdom of Great Britain and Northern Ireland GB 1 1 BARN 1996 1990-1999
Chad 47 Chad United Kingdom of Great Britain and Northern Ireland GB 1 1 BARN 1988 1980-1989
Chalice 48 Chalice United Kingdom of Great Britain and Northern Ireland GB 1 1 BARN 1995 1990-1999
Chariot 49 Chariot United Kingdom of Great Britain and Northern Ireland GB 1 1 BARN 1989 1980-1989
Chaser 50 Chaser United Kingdom of Great Britain and Northern Ireland GB 1 1 BARN 1997 1990-1999
Chevalier Tystofte 2 51 Chevalier Tystofte 2 United Kingdom of Great Britain and Northern Ireland GB 1 1 BARN 1830 1830-1959
Chieftain 52 Chieftain United Kingdom of Great Britain and Northern Ireland GB 1 1 BARN 1993 1990-1999
Chime 53 Chime United Kingdom of Great Britain and Northern Ireland GB 1 1 BARN 1997 1990-1999
Cilla 54 Cilla Sweden SE 1 1 BARN 1966 1960-1969
Claret 55 Claret United Kingdom of Great Britain and Northern Ireland GB 1 1 BARN 1980 1980-1989
Class 56 Class United Kingdom of Great Britain and Northern Ireland GB 1 1 BARN 2000 2000-2009
Cocktail 57 Cocktail United Kingdom of Great Britain and Northern Ireland GB 1 1 BARN 2003 2000-2009
Colada 58 Colada United Kingdom of Great Britain and Northern Ireland GB 1 1 BARN 1997 1990-1999
Concerto 59 Concerto United Kingdom of Great Britain and Northern Ireland GB 1 1 BARN 2006 2000-2009
Cooper 60 Cooper United Kingdom of Great Britain and Northern Ireland GB 1 1 BARN 1991 1990-1999
Cork 61 Cork United Kingdom of Great Britain and Northern Ireland GB 1 1 BARN 1992 1990-1999
Corniche 62 Corniche Germany DE 1 1 BARN 1983 1980-1989
Crusader AFP1290 63 Crusader AFP1290 United Kingdom of Great Britain and Northern Ireland GB 1 1 BARN 1995 1990-1999
Dallas 64 Dallas United Kingdom of Great Britain and Northern Ireland GB 1 BARN 1989 1980-1989
Dandy 65 Dandy United Kingdom of Great Britain and Northern Ireland GB 1 1 BARN 1984 1980-1989
Deba Abed 66 Deba Abed Denmark DK 1 1 BARN 1965 1960-1969
Decanter 67 Decanter United Kingdom of Great Britain and Northern Ireland GB 1 1 BARN 1996 1990-1999
Delibes 68 Delibes United Kingdom of Great Britain and Northern Ireland GB 1 1 BARN 1991 1990-1999
Delta AFP460 69 Delta AFP460 United Kingdom of Great Britain and Northern Ireland GB 1 1 BARN 1981 1980-1989
Derkado 70 Derkado Germany DE 1 1 BARN 1987 1980-1989
Diamant 71 Diamant Czech Republic CZ 1 1 BARN 1965 1960-1969
Digger 72 Digger United Kingdom of Great Britain and Northern Ireland GB 1 1 BARN 1983 1980-1989
Dina 73 Dina Denmark DK 1 1 BARN 1975 1970-1979
Domen 74 Domen Norway NO 1 1 BARN 1952 1830-1959
Drake 75 Drake Netherlands NL 1 1 BARN 1968 1960-1969
Drost 76 Drost Denmark DK 1 1 BARN 1954 1830-1959
Drum 77 Drum United Kingdom of Great Britain and Northern Ireland GB 1 1

BARN 2001 2000-2009
Egmont 78 Egmont United Kingdom of Great Britain and Northern Ireland GB 1 1 BARN 1980 1980-1989
Emir 79 Emir Netherlands NL 1 1 BARN 1962 1960-1969
Fairytale 80 Fairytale Denmark DK 1 1 BARN 2005 2000-2009
Favorit 81 Favorit Czech Republic CZ 1 1 BARN 1973 1970-1979
Forensic 82 Forensic United Kingdom of Great Britain and Northern Ireland GB 1 1 BARN 2006 2000-2009
Freja 83 Freja Sweden SE 1 1 BARN 1941 1830-1959
Georgie 84 Georgie United Kingdom of Great Britain and Northern Ireland GB 1 1 BARN 1973 1970-1979
Gitane 85 Gitane Netherlands NL 1 1 BARN 1976 1970-1979
Golden Promise 86 Golden Promise United Kingdom of Great Britain and Northern Ireland GB 1 1 BARN 1966 1960-1969
Golf 87 Golf United Kingdom of Great Britain and Northern Ireland GB 1 1 BARN 1980 1980-1989
Gull 88 Gull Sweden SE 1 1 BARN 1913 1830-1959
Gundel 89 Gundel Sweden SE 1 1 BARN 1984 1980-1989
Hannchen 90 Hannchen Sweden SE 1 1893 1830-1959
Hart 91 Hart United Kingdom of Great Britain and Northern Ireland GB 1 1 BARN 1986 1980-1989
Hellas 92 Hellas Sweden SE 1 1 BARN 1967 1960-1969
Helmi 93 Helmi Finland FI 1 1 BARN 1942 1830-1959
Hemingway 94 Hemingway United Kingdom of Great Britain and Northern Ireland GB 1 1 BARN 2012 2010-2017
Heron 95 Heron United Kingdom of Great Britain and Northern Ireland GB 1 1 BARN 1990 1990-1999
Husky AFP2429 96 Husky AFP2429 United Kingdom of Great Britain and Northern Ireland GB 1 1 BARN 2006 2000-2009
Imidis 97 Imidis 1 1 BARN 2006 2000-2009
Impala 98 Impala Netherlands NL 1 1 BARN 1964 1960-1969
Infinium 99 Infinium 1 1 BARN 2014 2010-2017
Ingrid 100 Ingrid Sweden SE 1 1 BARN 1956 1830-1959
Invictus 101 Invictus 1 1 BARN 2013 2010-2017
Isabella 102 Isabella Denmark DK 1 1 BARN 2004 2000-2009
Isaria 103 Isaria Germany DE 1 1 BARN 1924 1830-1959
Kassima 104 Kassima Netherlands NL 1 1 BARN 2003 2000-2009
Kenia 105 Kenia Denmark DK 1 1927 1830-1959
Klaxon 106 Klaxon United Kingdom of Great Britain and Northern Ireland GB 1 1 BARN 1981 1980-1989
Koral 107 Koral Czech Republic CZ 1 1 BARN 1980 1980-1989
Krona 108 Krona Germany DE 1 1990 1990-1999
Krystal 109 Krystal Czech Republic CZ 1 1 BARN 1981 1980-1989
KWS Irina 110 KWS Irina United Kingdom of Great Britain and Northern Ireland GB 1 BARN 2011 2010-2017
KWS Orphelia 111 KWS Orphelia United Kingdom of Great Britain and Northern Ireland GB 1 1 BARN 2010 2010-2017
KWS Vitara 112 KWS Vitara United Kingdom of Great Britain and Northern Ireland GB 1 1 BARN 2014 2010-2017
Kym 113 Kym United Kingdom of Great Britain and Northern Ireland GB 1 1 BARN 1979 1970-1979
Landlord 114 Landlord United Kingdom of Great Britain and Northern Ireland GB 1 1 BARN 1994 1990-1999
Lenta 115 Lenta Denmark DK 1 1 BARN 1943 1830-1959
Livet 116 Livet United Kingdom of Great Britain and Northern Ireland GB 1 1 BARN 1996 1990-1999

Lud 117 Lud United Kingdom of Great Britain and Northern Ireland GB 1 1 BARN 1976 1970-1979
Macaw 118 Macaw France FR 1 1 BARN 2002 2000-2009
Maja 119 Maja Denmark DK 1 1 BARN 1927 1830-1959
Mala Abed 120 Mala Abed Denmark DK 1 1 BARN 1972 1970-1979
Maresi 121 Maresi Germany DE 1 1 BARN 1986 1980-1989
Maris Mink 122 Maris Mink United Kingdom of Great Britain and Northern Ireland GB 1 1 BARN 1973 1970-1979
Maypole 123 Maypole United Kingdom of Great Britain and Northern Ireland GB 1 1 BARN 2000 2000-2009
Meltan 124 Meltan Sweden SE 1 1 BARN 1990 1990-1999
Midas 125 Midas United Kingdom of Great Britain and Northern Ireland GB 1 1 BARN 1970 1970-1979
Momentum 126 Momentum United Kingdom of Great Britain and Northern Ireland GB 1 1 BARN 2012 2010-2017
Natasha 127 Natasha France FR 1 1 BARN 1982 1980-1989
Natasia 128 Natasia United Kingdom of Great Britain and Northern Ireland GB 1 1 BARN 2010 2010-2017
Nomad 129 Nomad United Kingdom of Great Britain and Northern Ireland GB 1 1 BARN 1987 1980-1989
Nordal 130 Nordal Denmark DK 1 1 BARN 1971 1970-1979
Novello 131 Novello United Kingdom of Great Britain and Northern Ireland GB 1 1 BARN 2000 2000-2009
Odyssey 132 Odyssey France FR 1 1 BARN 2009 2000-2009
Okos 133 Okos Italy IT 1 1 BARN 1975 1970-1979
Olympus 134 Olympus 1 1 BARN 2013 2010-2017
Optic 135 Optic United Kingdom of Great Britain and Northern Ireland GB 1 1 BARN 1992 1990-1999
Orbit 136 Orbit Slovakia SK 1 1 BARN 1986 1980-1989
Otto 137 Otto Italy IT 1 1 BARN 1972 1970-1979
Overture 138 Overture France FR 1 1 BARN 2009 2000-2009
Paloma 139 Paloma Denmark DK 1 1 BARN 1996 1990-1999
Pewter 140 Pewter United Kingdom of Great Britain and Northern Ireland GB 1 1 BARN 1998 1990-1999
Pitcher 141 Pitcher United Kingdom of Great Britain and Northern Ireland GB 1 1 BARN 1993 1990-1999
Poker 142 Poker United Kingdom of Great Britain and Northern Ireland GB 1 1 BARN 2003 2000-2009
Potter 143 Potter Sweden SE 1 1 BARN 1997 1990-1999
Power 144 Power Denmark DK 1 BARN 2002 2000-2009
Prague 145 Prague United Kingdom of Great Britain and Northern Ireland GB 1 1 BARN 2004 2000-2009
Prestige 146 Prestige United Kingdom of Great Britain and Northern Ireland GB 1 1 BARN 1998 1990-1999
Prisma 147 Prisma Netherlands NL 1 1 BARN 1985 1980-1989
Proctor 148 Proctor United Kingdom of Great Britain and Northern Ireland GB 1 BARN 1952 1830-1959
Prodigal 149 Prodigal United Kingdom of Great Britain and Northern Ireland GB 1 1 BARN 2010 2010-2017
Propino 150 Propino United Kingdom of Great Britain and Northern Ireland GB 1 1 BARN 2007 2000-2009
Publican 151 Publican United Kingdom of Great Britain and Northern Ireland GB 1 1 BARN 2004 2000-2009
Quench 152 Quench United Kingdom of Great Britain and Northern Ireland GB 1 1 BARN 2004 2000-2009
Rainbow 153 Rainbow United Kingdom of Great Britain and Northern Ireland GB

1 1 BARN 1994 1990-1999
Rapid 154 Rapid Czech Republic CZ 1 1 BARN 1976 1970-1979
Reggae 155 Reggae Netherlands NL 1 1 BARN 1992 1990-1999
Renaissance 156 Renaissance 1 1 BARN 2011 2010-2017
Renata 157 Renata United Kingdom of Great Britain and Northern Ireland GB 1 1 BARN 1994 1990-1999
RGT Conquest 158 RGT Conquest United Kingdom of Great Britain and Northern Ireland GB 1 1 BARN 2013 2010-2017
RGT Planet 159 RGT Planet United Kingdom of Great Britain and Northern Ireland GB 1 1 BARN 2014 2010-2017
Rhynchostar 160 Rhynchostar United Kingdom of Great Britain and Northern Ireland GB 1 1 BARN 2010 2010-2017
Ria 161 Ria Germany DE 1 1 BARN 1987 1980-1989
Rika 162 Rika Sweden SE 1 BARN 1949 1830-1959
Riviera 163 Riviera United Kingdom of Great Britain and Northern Ireland GB 1 1 BARN 1992 1990-1999
Romi 164 Romi Denmark DK 1 1 BARN 1983 1980-1989
Rummy 165 Rummy United Kingdom of Great Britain and Northern Ireland GB 1 1 BARN 2003 2000-2009
Salka 166 Salka Denmark DK 1 1 BARN 1973 1970-1979
Saloon 167 Saloon United Kingdom of Great Britain and Northern Ireland GB 1 1 BARN 1997 1990-1999
Salve 168 Salve Sweden SE 1 1 BARN 1974 1970-1979
Scandium 169 Scandium Denmark DK 1 1 BARN 2004 2000-2009
Scarlett 170 Scarlett Germany DE 1 1 BARN 1995 1990-1999
Sebastian 171 Sebastian Denmark DK 1 1 BARN 2000 2000-2009
Senat 172 Senat Sweden SE 1 1 BARN 1974 1970-1979
Simba 173 Simba Denmark DK 1 1 BARN 2003 2000-2009
Simon 174 Simon Sweden SE 1 1 BARN 1978 1970-1979
Skittle 175 Skittle United Kingdom of Great Britain and Northern Ireland GB 1 1 BARN 2003 2000-2009
Spartan 176 Spartan Czech Republic CZ 1 1 BARN 1977 1970-1979
Spey 177 Spey United Kingdom of Great Britain and Northern Ireland GB 1 BARN 1995 1990-1999
Spire 178 Spire United Kingdom of Great Britain and Northern Ireland GB 1 1 BARN 1999 1990-1999
Starlight 179 Starlight United Kingdom of Great Britain and Northern Ireland GB 1 1 BARN 1997 1990-1999
Static 180 Static United Kingdom of Great Britain and Northern Ireland GB 1 1 BARN 1996 1990-1999
Steffi 181 Steffi Germany DE 1 1 BARN 1989 1980-1989
Steina 182 Steina Germany DE 1 1 BARN 1980 1980-1989
Stendes 183 Stendes Latvia LV 1 1 BARN 1972 1970-1979
Sultan 184 Sultan Netherlands NL 1 1 BARN 1966 1960-1969
Summit 185 Summit United Kingdom of Great Britain and Northern Ireland GB 1 1 BARN 2008 2000-2009
SW Scania 186 SW Scania Sweden SE 1 1 BARN 2002 2000-2009
SY Taberna 187 SY Taberna United Kingdom of Great Britain and Northern Ireland GB 1 1 BARN 2008 2000-2009
SY Universal 188 SY Universal United Kingdom of Great Britain and Northern Ireland GB 1 1 BARN 2009 2000-2009
Tankard 189 Tankard United Kingdom of Great Britain and Northern Ireland GB 1 1 BARN 1993 1990-1999
Taphouse 190 Taphouse United Kingdom of Great Britain and Northern Ireland GB 1 1 BARN 2004 2000-2009
Tartan 191 Tartan United Kingdom of Great Britain and Northern Ireland GB 1

1 BARN 2004 2000-2009
Tavern 192 Tavern United Kingdom of Great Britain and Northern Ireland GB 1 1 BARN 1997 1990-1999
Tennis 193 Tennis United Kingdom of Great Britain and Northern Ireland GB 1 1 BARN 1984 1980-1989
Tocada 194 Tocada Germany DE 1 1 BARN 2002 2000-2009
Tremois 195 Tremois France FR 1 1 BARN 1989 1980-1989
Trinity 196 Trinity United Kingdom of Great Britain and Northern Ireland GB 1 1 BARN 1993 1990-1999
Triumph 197 Triumph Germany DE 1 1 BARN 1973 1970-1979
Troubadour 198 Troubadour France FR 1 1 BARN 1982 1980-1989
Tweed 199 Tweed United Kingdom of Great Britain and Northern Ireland GB 1 1 BARN 1980 1980-1989
Tyne 200 Tyne United Kingdom of Great Britain and Northern Ireland GB 1 1 BARN 1985 1980-1989
Union 201 Union Germany DE 1 1 BARN 1955 1830-1959
Vada 202 Vada Netherlands NL 1 1 BARN 1958 1830-1959
Valticky 203 Valticky Czech Republic CZ 1 1 BARN 1950 1830-1959
Vankkuri 204 Vankkuri Finland FI 1 1 BARN 1943 1830-1959
Velvet 205 Velvet 1 1 BARN 2001 2000-2009
Villa 206 Villa Germany DE 1 1 BARN 1968 1960-1969
Volla 207 Volla Germany DE 1 1 BARN 1957 1830-1959
Waggon 208 Waggon United Kingdom of Great Britain and Northern Ireland GB 1 1 BARN 2002 2000-2009
Westminster 209 Westminster United Kingdom of Great Britain and Northern Ireland GB 1 1 BARN 2002 2000-2009
Wing 210 Wing Sweden SE 1 1 BARN 1968 1960-1969
Wisa 211 Wisa Germany DE 1 1 BARN 1951 1830-1959
Zephyr 2RSB 212 Zephyr 2RSB Netherlands NL 1 1 BARN 1965 1960-1969
92.25 213 92.25 United Kingdom of Great Britain and Northern Ireland GB Unknown
14029 214 14029 Germany DE Unknown
88513 215 88513 Sweden SE Unknown
413287 216 413287 Unknown
(343/6 x 34/6) x J-427 217 (343/6 x 34/6) x J-427 Unknown
LBW6153 P40 218 LBW6153 P40 Unknown
ML-I x LP2.51784 219 ML-I x LP2.51784 Unknown
11719/59 220 11719/59 Unknown
1208/67 221 1208/67 Unknown
1293/70 222 1293/70 Unknown
22114/84M 223 22114/84M Unknown
26744/84 224 26744/84 Unknown
22746Co41 225 22746Co41 United Kingdom of Great Britain and Northern Ireland GB Unknown
TSS 311-54 226 TSS 311-54 United Kingdom of Great Britain and Northern Ireland GB Unknown
36462 x 14008/64 227 36462 x 14008/64 Unknown
49113-502-11 228 49113-502-11 Unknown
49428/69 229 49428/69 Unknown
678 x Ub.Baco 230 678 x Ub.Baco Unknown
84160.1.3.3 231 84160.1.3.3 Germany DE Unknown
Abed 079 232 Abed 079 Denmark DK Unknown
Abed 3371 233 Abed 3371 Denmark DK Unknown
Abed Denso 234 Abed Denso Denmark DK Unknown
Acclaim 235 Acclaim Germany DE 1984 1980-1989
African landvariety 236 African landvariety Unknown

Agio 237 Agio Netherlands NL   1950 1830-1959
Akka 238 Akka Sweden SE   1970 1970-1979
Alis 239 Alis Denmark DK   1985 1980-1989
All 3109 240 All 3109   Unknown
Alsa 241 Alsa Germany DE   1963 1960-1969
Amazone 242 Amazone Germany DE   1986 1980-1989
Amber 243 Amber United Kingdom of Great Britain and Northern Ireland GB
1992 1990-1999
Amsel 244 Amsel Germany DE   1960 1960-1969
Arabische 245 Arabische   Unknown
Ares 246 Ares France FR   1959 1830-1959
Ariel 247 Ariel Sweden SE   1987 1980-1989
Arivat 248 Arivat United States of America US   1940 1830-1959
Arla 249 Arla Sweden SE   1962 1960-1969
Asplund 250 Asplund Sweden SE   1910 1830-1959
Astrix 251 Astrix France FR   1969 1960-1969
Atem 252 Atem Netherlands NL   1980 1980-1989
Aufis 253 Aufis Germany DE   1974 1970-1979
B 2145 254 B 2145   Unknown
Bartok 255 Bartok United Kingdom of Great Britain and Northern Ireland GB
Unknown
Bavaria 256 Bavaria Germany DE   1910 1830-1959
Bavarian landvariety 257 Bavarian landvariety Germany DE   Unknown
Bethges II 258 Bethges II Germany DE   Unknown
Bethges III 259 Bethges III Germany DE   Unknown
Bethges XIII 260 Bethges XIII Germany DE   Unknown
Bordia 261 Bordia Belgium BE   1924 1830-1959
Brahms 262 Brahms United Kingdom of Great Britain and Northern Ireland GB
1995 1990-1999
Breun 1453e16 x Him.T253 263 Breun 1453e16 x Him.T253 Germany DE
Unknown
Breun 1622 264 Breun 1622 Germany DE   Unknown
Breun 1747 265 Breun 1747 Germany DE   Unknown
Breun 3556a 266 Breun 3556a Germany DE   Unknown
Britannia 267 Britannia United Kingdom of Great Britain and Northern
Ireland GB   1989 1980-1989
Cambrinus 268 Cambrinus Netherlands NL   1962 1960-1969
Carlsberg II 269 Carlsberg II Denmark DK   1953 1830-1959
Carnival 270 Carnival United Kingdom of Great Britain and Northern Ireland
GB   1981 1980-1989
Casino 271 Casino United Kingdom of Great Britain and Northern Ireland GB
1987 1980-1989
Cask 272 Cask United Kingdom of Great Britain and Northern Ireland GB
1992 1990-1999
Cebeco 6721 x L100 273 Cebeco 6721 x L100 Netherlands NL   Unknown
Cebeco 7931 x Pompadour 274 Cebeco 7931 x Pompadour Netherlands NL
Unknown
Cebeco 6801 x GB 1605 275 Cebeco 6801 x GB 1605 Netherlands NL   Unknown
Cebeco 8331 276 Cebeco 8331 Netherlands NL   Unknown
Celechovicky hanacky 277 Celechovicky hanacky Czech Republic CZ   1956
1830-1959
Ceres 278 Ceres France FR   1962 1960-1969
CI 1237 279 CI 1237   Unknown
CJV 1483 280 CJV 1483   Unknown
Clara 281 Clara Sweden SE   1964 1960-1969
Clermont 282 Clermont France FR   1967 1960-1969

Colston 283 Colston United Kingdom of Great Britain and Northern Ireland GB 2000 2000-2009
Columbus 284 Columbus Denmark DK 2010 2010-2017
Conchita 285 Conchita Germany DE 2007 2000-2009
CPBT B55 286 CPBT B55 Unknown
Cropton 287 Cropton United Kingdom of Great Britain and Northern Ireland GB 2006 2000-2009
CSBA 1096/1022 288 CSBA 1096/1022 Unknown
D 80-20 289 D 80-20 Unknown
Dana 290 Dana Denmark DK 1964 1960-1969
Danubia 291 Danubia Germany DE 1912 1830-1959
Defra 293 Defra Germany DE 1984 1980-1989
Denar 294 Denar Czech Republic CZ 1969 1960-1969
Dera 295 Dera Germany DE 1982 1980-1989
DN7 296 DN7 Unknown
Donaria 297 Donaria Germany DE 1941 1830-1959
Dram 298 Dram United Kingdom of Great Britain and Northern Ireland GB 1977 1970-1979
Dray 299 Dray United Kingdom of Great Britain and Northern Ireland GB 1997 1990-1999
Dwarf 300 Dwarf United Kingdom of Great Britain and Northern Ireland GB Unknown
Earl 301 Earl United Kingdom of Great Britain and Northern Ireland GB 1947 1830-1959
Ekonom 302 Ekonom Czech Republic CZ 1960 1960-1969
Elbo 303 Elbo Denmark DK 1968 1960-1969
Elsa 304 Elsa France FR 1980 1980-1989
Erica 305 Erica Unknown
Erna 306 Erna Netherlands NL 1981 1980-1989
ET 181 x Kara x Halcyon 307 ET 181 x Kara x Halcyon Unknown
Etna 308 Etna Denmark DK 1991 1990-1999
Extract 309 Extract United Kingdom of Great Britain and Northern Ireland GB 1997 1990-1999
F 784-70/3 310 F 784-70/3 Unknown
Fabel 311 Fabel Unknown
Fergie 312 Fergie United Kingdom of Great Britain and Northern Ireland GB 1990 1990-1999
Firl. 621 313 Firl. 621 Germany DE Unknown
Flare 314 Flare United Kingdom of Great Britain and Northern Ireland GB 1980 1980-1989
Fleet 315 Fleet United Kingdom of Great Britain and Northern Ireland GB 1985 1980-1989
Flute 316 Flute United Kingdom of Great Britain and Northern Ireland GB 1997 1990-1999
Force 317 Force Unknown
Fractal 318 Fractal United Kingdom of Great Britain and Northern Ireland GB 1996 1990-1999
Frisia 319 Frisia Germany DE 1955 1830-1959
Galina 320 Galina Germany DE 1972 1970-1979
Gamma-Ray Mutant 321 Gamma-Ray Mutant United Kingdom of Great Britain and Northern Ireland GB Unknown
Georgine 322 Georgine Germany DE 1932 1830-1959
Gerbel 323 Gerbel France FR 1977 1970-1979
Gerlinde 324 Gerlinde Germany DE 1979 1970-1979
Gimpel 325 Gimpel Germany DE 1979 1970-1979
Goldie 326 Goldie Sweden SE 1992 1990-1999

Goldmarker 327 Goldmarker United Kingdom of Great Britain and Northern Ireland GB 1977 1970-1979
Gotland landvariety 328 Gotland landvariety Sweden SE Unknown
Grand Prix 2 329 Grand Prix 2 Netherlands NL Unknown
Grit 330 Grit Germany DE 1979 1970-1979
Gull Mutant 331 Gull Mutant Sweden SE Unknown
Gunilla 332 Gunilla Sweden SE 1970 1970-1979
H 204 333 H 204 Unknown
Hordeum laevigatum 334 Hordeum laevigatum Unknown
Hordeum deficiens x Sergeant 335 Hordeum deficiens x Sergeant Unknown
Hadm. 46459-68 336 Hadm. 46459-68 Germany DE Unknown
Hadm. 46619-68 337 Hadm. 46619-68 Germany DE Unknown
Hadm. 46655 338 Hadm. 46655 Germany DE Unknown
Hadm. 46813-68 339 Hadm. 46813-68 Germany DE Unknown
Hadm. 55474-67 x Derenburg 480-68 340 Hadm. 55474-67 x Derenburg 480-68 Germany DE Unknown
Hadm. 55648-85 341 Hadm. 55648-85 Germany DE Unknown
Hado 342 Hado Czech Republic CZ 1907 1830-1959
Hadostreng 343 Hadostreng Germany DE 1907 1830-1959
Haha 344 Haha Germany DE 1941 1830-1959
Haisa 345 Haisa Germany DE 1939 1830-1959
Hana 346 Hana Czech Republic CZ 1973 1970-1979
Hauter x Hatif de Grignon 347 Hauter x Hatif de Grignon France FR Unknown
HB 820-12 348 HB 820-12 Unknown
Heine 05530 349 Heine 05530 Germany DE Unknown
Heine 2149 350 Heine 2149 Germany DE Unknown
Heine 4242 351 Heine 4242 Germany DE Unknown
Heine 4808 352 Heine 4808 Germany DE Unknown
Heines Hanna 353 Heines Hanna Germany DE Unknown
Henley 354 Henley France FR 2006 2000-2009
Henni 355 Henni Germany DE 1995 1990-1999
Heritage 356 Heritage United Kingdom of Great Britain and Northern Ireland GB Unknown
Herta 357 Herta Sweden SE 1949 1830-1959
Hind 358 Hind France FR 1996 1990-1999
Ho. 4595/51 359 Ho. 4595/51 Unknown
Hood 360 Hood United Kingdom of Great Britain and Northern Ireland GB 1978 1970-1979
HP 5466 361 HP 5466 Unknown
HVS 18707 362 HVS 18707 Unknown
HVS 2.1142-4-79 363 HVS 2.1142-4-79 Unknown
Imperial 364 Imperial Unknown
Indian landrace 365 Indian landrace India IN Unknown
Indian landvariety 366 Indian landvariety India IN Unknown
J25 367 J25 Unknown
Joline 368 Joline United Kingdom of Great Britain and Northern Ireland GB 1988 1980-1989
Julia 369 Julia Netherlands NL 1968 1960-1969
Juno 370 Juno United Kingdom of Great Britain and Northern Ireland GB 1994 1990-1999
K 829 x Ariel 371 K 829 x Ariel Unknown
Karat 372 Karat Czech Republic CZ 1981 1980-1989
Katy 373 Katy France FR 1971 1970-1979
KM 1192 374 KM 1192 Czech Republic CZ Unknown
Kneifel 375 Kneifel Czech Republic CZ 1926 1830-1959

Korinna 376 Korinna Germany DE 1988 1980-1989
Kristina 377 Kristina Sweden SE 1968 1960-1969
Lada 378 Lada Germany DE 1979 1970-1979
landvariety 379 landvariety Unknown
Libelle 380 Libelle Germany DE 1990 1990-1999
Lignee 207 381 Lignee 207 France FR 1962 1960-1969
Lignee 2730E 382 Lignee 2730E France FR Unknown
Linden 383 Linden United Kingdom of Great Britain and Northern Ireland GB 1998 1990-1999
Lofa 384 Lofa Denmark DK 1968 1960-1969
Lome 385 Lome Unknown
LP 426.92 x LP 6800.92 386 LP 426.92 x LP 6800.92 Unknown
LP 629.1-95 387 LP 629.1-95 Unknown
Luke 388 Luke United Kingdom of Great Britain and Northern Ireland GB 1976 1970-1979
Lux 389 Lux Denmark DK 1997 1990-1999
Lyallpur 390 Lyallpur Pakistan PK Unknown
Maga 391 Maga Unknown
Magnif 104 392 Magnif 104 United Kingdom of Great Britain and Northern Ireland GB Unknown
Magnif 105 393 Magnif 105 United Kingdom of Great Britain and Northern Ireland GB Unknown
Magnum 394 Magnum United Kingdom of Great Britain and Northern Ireland GB 1980 1980-1989
Mari 395 Mari Sweden SE 1960 1960-1969
Marina 396 Marina Germany DE 1990 1990-1999
Marion 397 Marion France FR 1981 1980-1989
Maris Bulbeck 398 Maris Bulbeck United Kingdom of Great Britain and Northern Ireland GB Unknown
Maris Yak x W 1001 399 Maris Jak x W 1001 United Kingdom of Great Britain and Northern Ireland GB Unknown
Maskin 400 Maskin Norway NO 1918 1830-1959
Maud 401 Maud Sweden SE 1991 1990-1999
Maythorpe 402 Maythorpe United Kingdom of Great Britain and Northern Ireland GB 1954 1830-1959
Miln 155/38 403 Miln 155/38 Unknown
Minerva 404 Minerva Netherlands NL 1955 1830-1959
Minstrel 406 Minstrel 2002 2000-2009
Monarch 407 Monarch Unknown
Monte Cristo 408 Monte Cristo India IN Unknown
Moosburger Rhatia 409 Moosburger Rhatia Germany DE 1913 1830-1959
Morgenrot 410 Morgenrot Germany DE 1944 1830-1959
Mutant SS 55 411 Mutant SS 55 Unknown
Nackta 412 Nackta Germany DE 1969 1960-1969
NFC 461181 413 NFC 461181 United Kingdom of Great Britain and Northern Ireland GB Unknown
NFC 497-12 414 NFC 497-12 United Kingdom of Great Britain and Northern Ireland GB Unknown
NFC 499-69 415 NFC 499-69 United Kingdom of Great Britain and Northern Ireland GB Unknown
NFC 514-5 416 NFC 514-5 United Kingdom of Great Britain and Northern Ireland GB Unknown
NFC 5563 417 NFC 5563 United Kingdom of Great Britain and Northern Ireland GB Unknown
NFC 85/1/3 418 NFC 85/1/3 United Kingdom of Great Britain and Northern Ireland GB Unknown

NFC 94-11 419 NFC 94-11 United Kingdom of Great Britain and Northern Ireland GB Unknown
NFC 94-20 420 NFC 94-20 United Kingdom of Great Britain and Northern Ireland GB Unknown
NFC 94-4 421 NFC 94-4 United Kingdom of Great Britain and Northern Ireland GB Unknown
NFC Tipple 422 NFC Tipple United Kingdom of Great Britain and Northern Ireland GB 2005 2000-2009
NFC1983-27 423 NFC1983-27 United Kingdom of Great Britain and Northern Ireland GB Unknown
NFC86/60 424 NFC86/60 United Kingdom of Great Britain and Northern Ireland GB Unknown
Nolcuv A or Dregerev Imp 425 Nolcuv A or Dregerev Imp Unknown
NSL 95-2949 426 NSL 95-2949 United Kingdom of Great Britain and Northern Ireland GB Unknown
NSL 97-5547 427 NSL 97-5547 United Kingdom of Great Britain and Northern Ireland GB Unknown
NSL07-8124-A 428 NSL07-8124-A United Kingdom of Great Britain and Northern Ireland GB Unknown
Olli 429 Olli Finland FI 1927 1830-1959
Opal 430 Opal Denmark DK 1922 1830-1959
Orcivale 431 Orcivale France FR 1994 1990-1999
Oriol 432 Oriol Germany DE 1969 1960-1969
Orthega 433 Orthega Germany DE 1996 1990-1999
Ortoli 434 Ortoli United Kingdom of Great Britain and Northern Ireland GB 1999 1990-1999
Otira 435 Otira United Kingdom of Great Britain and Northern Ireland GB 1998 1990-1999
Oxbridge 436 Oxbridge United Kingdom of Great Britain and Northern Ireland GB 2003 2000-2009
Panda 437 Panda France FR 1983 1980-1989
Pasadena 438 Pasadena Germany DE 1998 1990-1999
Peel 439 Peel United Kingdom of Great Britain and Northern Ireland GB 1992 1990-1999
Peroga 440 Peroga Germany DE 1962 1960-1969
PF 52213 441 PF 52213 Unknown
Piccolo 442 Piccolo Netherlands NL Unknown
Picnic 443 Picnic Unknown
Pikkio 444 Pikkio Unknown
Pirol 445 Pirol Germany DE 1950 1830-1959
Piroline 446 Piroline Germany DE 1953 1830-1959
Platoon 447 Platoon United Kingdom of Great Britain and Northern Ireland GB 1993 1990-1999
Plumage Archer 448 Plumage Archer United Kingdom of Great Britain and Northern Ireland GB Unknown
PP79 449 PP79 Unknown
Prentice 450 Prentice Denmark DK Unknown
Prolog 451 Prolog Germany DE 2000 2000-2009
Proskowetz Hanna 452 Proskowetz Hanna Czech Republic CZ 1884 1830-1959
Ragusa 453 Ragusa Germany DE 1929 1830-1959
Recept 454 Recept Unknown
Regatta 455 Regatta United Kingdom of Great Britain and Northern Ireland GB 1987 1980-1989
Regent 456 Regent United Kingdom of Great Britain and Northern Ireland GB 1981 1980-1989
Riegel 457 Riegel Unknown

Rigel 458 Rigel Denmark DK 1944 1830-1959
Rosie Abed 459 Rosie Abed Denmark DK 1980 1980-1989
Roxana 460 Roxana Germany DE 2000 2000-2009
RPB 16/71 461 RPB 16/71 United Kingdom of Great Britain and Northern Ireland GB Unknown
RPB 713/77 462 RPB 713/77 United Kingdom of Great Britain and Northern Ireland GB Unknown
Rupal 463 Rupal Sweden SE 1972 1970-1979
Rupee 464 Rupee India IN Unknown
S3170/Abyss 465 S3170/Abyss Unknown
S-487 466 S-487 Unknown
S77323 467 S77323 Unknown
Salome 468 Salome Germany DE 1981 1980-1989
Sandra 469 Sandra France FR 1975 1970-1979
Sarah 470 Sarah Germany DE 1998 1990-1999
Saxo 471 Saxo Sweden SE 1993 1990-1999
Scanian Barley 472 Scanian Barley Unknown
SCRI 8313 473 SCRI 8313 United Kingdom of Great Britain and Northern Ireland GB Unknown
Seger 474 Seger Sweden SE 1926 1830-1959
Sherpa 475 Sherpa United Kingdom of Great Britain and Northern Ireland GB Unknown
SJ 028126 476 SJ 028126 Denmark DK Unknown
SJ 050549 477 SJ 050549 Denmark DK Unknown
SJ 203256 478 SJ 203256 Denmark DK Unknown
SJ 930331 479 SJ 930331 Denmark DK Unknown
SJ 96-1441 480 SJ 96-1441 Denmark DK Unknown
Sk 783 x Ce Dc-74 481 Sk 783 x Ce Dc-74 Unknown
St434/62 482 St434/62 Unknown
Stamm 483 Stamm Germany DE Unknown
Stamm 101 484 Stamm 101 Germany DE Unknown
Stamm 210 485 Stamm 210 Germany DE Unknown
Stanka 486 Stanka Germany DE 1954 1830-1959
Stanza 487 Stanza United Kingdom of Great Britain and Northern Ireland GB 1988 1980-1989
Steward x NFC 406/113 488 Steward x NFC 406/113 United Kingdom of Great Britain and Northern Ireland GB Unknown
Streng Franken III 489 Streng Franken III Germany DE 1948 1830-1959
Sulu 490 Sulu Australia AU 1957 1830-1959
Svalof Hanna 491 Svalof Hanna Sweden SE Unknown
Svanhals 492 Svanhals Sweden SE 1903 1830-1959
SW 89692 493 SW 89692 Sweden SE Unknown
Swallow 494 Swallow United Kingdom of Great Britain and Northern Ireland GB 1960 1960-1969
Tammi 495 Tammi Finland FI 1937 1830-1959
Tamtam 496 Tamtam Australia AU Unknown
TCE 141 497 TCE 141 Unknown
Tellus 498 Tellus Sweden SE 1970 1970-1979
Trebon 499 Trebon Sweden SE 1994 1990-1999
Triple awn lemma 500 Triple awn lemma Unknown
Troon 501 Troon United Kingdom of Great Britain and Northern Ireland GB 2002 2000-2009
Troop 502 Troop Unknown
Universe 503 Universe United Kingdom of Great Britain and Northern Ireland GB 1973 1970-1979
V 813 504 V 813 Unknown

Valticky B 505 Valticky B Czech Republic CZ Unknown
Varberg 506 Varberg Germany DE 2006 2000-2009
Vaughn 507 Vaughn United States of America US Unknown
VDH 233-79 508 VDH 233-79 Unknown
Vega 509 Vega Denmark DK 1976 1970-1979
Viskosa 510 Viskosa Germany DE 1999 1990-1999
Vitesse 511 Vitesse Unknown
Voldagsen 512 Voldagsen Germany DE Unknown
Vortex 513 Vortex 2000 2000-2009
VSB 10-15 x NFC 1440/80 514 VSB 10-15 x NFC 1440/80 Unknown
Weib. 5973 x Weib. 5853 515 Weib. 5973 x Weib. 5853 Germany DE Unknown
Weihenstephaner MR 1 516 Weihenstephaner MR 1 Germany DE 1935 1830-1959
Weihenstephaner MR 2 517 Weihenstephaner MR 2 Germany DE 1939 1830-1959
Wicket 518 Wicket United Kingdom of Great Britain and Northern Ireland GB 2002 2000-2009
Wong 519 Wong China CN Unknown
Woodcock 520 Woodcock Unknown
Ymer 521 Ymer Sweden SE 1944 1830-1959
Zeppelin 522 Zeppelin Unknown
Goldthorpe 523 Goldthorpe United Kingdom of Great Britain and Northern Ireland GB 1889 1830-1959
Tern 524 Tern Germany DE 1965 1960-1969
MGH 61229 525 MGH 61229 Netherlands NL Unknown
MGH 6271 526 MGH 6271 Netherlands NL Unknown
Hadm. 96677-87 527 Hadm. 96677-87 Germany DE Unknown
TS42/3/5 528 TS42/3/5 Unknown
BH4/200/5/90 529 BH4/200/5/90 Unknown
Z90-552 530 Z90-552 Unknown
Atlas USA 531 Atlas USA United States of America US 1924 1830-1959
Brioni 532 Brioni 1 2011 2010-2017
Opal B 533 Opal B Unknown
Breun 4714 534 Breun 4714 Germany DE Unknown
Genie 535 Genie 2009 2000-2009
Tesla 536 Tesla 2010 2010-2017
Sk 783 537 Sk 783 Unknown
Ce Dc-74 538 Ce Dc-74 Unknown
Havanna 539 Havanna Unknown
Marnie 540 Marnie Unknown
3192 f 56 541 3192 f 56 Unknown
Doyen 542 Doyen United Kingdom of Great Britain and Northern Ireland GB 1 2001 2000-2009
Mona 543 Mona Sweden SE 1 1970 1970-1979
Pallas 544 Pallas Sweden SE 1958 1830-1959
Vivaldi 545 Vivaldi Unknown
NSL04-4299-B 546 NSL04-4299-B Unknown
PF14 547 PF14 Unknown
Corgi 548 Corgi United Kingdom of Great Britain and Northern Ireland GB 1985 1980-1989
15533 Co 549 15533 Co Unknown
Crusader 550 Crusader Netherlands NL 1976 1970-1979
Delta 551 Delta Netherlands NL 1959 1830-1959
Tyra 552 Tyra Denmark DN 1975 1970-1979
Algerian x Herta 8 553 (Algerian x Herta 8) Unknown
Graphic 554 Graphic Unknown
